# Influence of Solid Filler on the Rheological Properties of Propellants Based on Energetic Thermoplastic Elastomer

**DOI:** 10.3390/ma16020808

**Published:** 2023-01-13

**Authors:** Jing Zhang, Zhen Wang, Shixiong Sun, Yunjun Luo

**Affiliations:** 1 School of Materials Science and Technology, Beijing Institute of Technology, Beijing 100081, China; 2 Key Laboratory for Ministry of Education of High Energy Density Materials, Beijing 100081, China; 3 School of Chemistry and Chemical Engineering, North University of China, Taiyuan 030051, China; 4 Dezhou Industrial Technology Research Institute of North University of China, Dezhou 253034, China

**Keywords:** energetic thermoplastic elastomer (ETPE), solid filler, rheological property

## Abstract

Glycidyl azide polymer-energetic thermoplastic elastomer propellant (GAP-ETPE) has high development prospects as a green solid propellant, although the preparation of GAP-ETPE with excellent performance is still a challenge. Focusing on the demand of high-strength solid propellants for free-loading rocket motors, a GAP-ETPE model propellant with excellent overall performance was prepared in this work, and the influence of adhesive structure characteristics on its fluidity was studied. Furthermore, the influence of filler on the rheological properties of the model propellant was investigated by introducing hexogen (RDX) and Al, and a corresponding two-phase model was established. The results may provide a reference for the structural design, molding process, and parameter selection of high-performance GAP-based green solid propellants.

## 1. Introduction

A solid propellant is a kind of composite energetic material which provides thrust energy for solid rocket engines. According to the characteristics of adhesive structure, solid propellants could be divided into thermosetting solid propellants and thermoplastic solid propellants [1]. However, the adhesive in thermosetting propellants could promote the formation of chemical crosslinking networks which prevent the recycling of propellants. Moreover, the oxidant used in the process, ammonium perchlorate (AP), could decompose into HCl, causing environmental pollution. Although thermoplastic propellants have the possibility of being recycled, the existing thermoplastic solid propellants are mainly modified double-base propellants which contain amounts of highly sensitive nitroglycerin (NG), resulting in risks during recycling. Accordingly, this type of propellant is often incinerated after the expiration of service, resulting in waste of resources, while the process is also dangerous. Therefore, recyclable green solid propellants have become one of the research focuses in recent years [2,3,4,5,6].

Green solid propellants have the characteristics of low toxicity, savings in energy and environmental processes, use of recyclable raw materials and samples, and clean gas [6]. The main research directions of such propellants focus on the development of lead-free double-base propellants [7], reusable energetic thermoplastic elastomer propellants [8], and green composite solid propellants [9]. Among them, the research on lead-free double-base propellants and green composite solid propellants mainly focuses on the use of clean gas, but the difficulty of reuse has not improved. Energetic thermoplastic elastomer propellants (ETPE) are linear polymers obtained by introducing energetic groups (such as -NO_2_; -ONO_2_; -N_3_; -NF_2_ and -NNO_2_) into thermoplastic elastomers, which combines the characteristics of the thermoplastic elastomers and energetic materials [10]. Propellants based on energetic thermoplastic elastomers have 3R characteristics (recycle, recover, reuse), which is an important research direction for green solid propellants [11]. Among this type of propellant, glycidyl azide polymer-energetic thermoplastic elastomer propellant (GAP-ETPE) is the most promising energetic thermoplastic elastomer due to its high energy level, excellent mechanical properties, clean gas, and relatively mature process [12]. Therefore, it is of great significance to explore the processing technology of GAP-based energetic thermoplastic elastomer propellants.

In our previous study, GAP-ETPE with excellent properties was synthesized and its flow-characteristics were explored. Focusing on the demand of high strength solid propellants for free-loading rocket motors, ETPE/NC/NENA adhesive system based on GAP-ETPE was designed, and the influence of adhesive structure on its fluidity was studied. Furthermore, the influence of filler on the rheological properties of the model propellant was investigated by introducing hexogen (RDX) and Al, and a corresponding two-phase model was established. The results may provide a reference for the structural design, molding process and parameter selection of high-performance GAP-based green solid propellants.

## 2. Materials and Methods

The raw materials of the model propellant were blended in an open two-roll milling machine at 70 °C. Then, the blends were tableted by flat-panel curing at 70 °C. The samples (listed in Table 1) were named after their powder content (e.g., Al-10, Al-20, Al-30, and Al-40 refer to the samples containing 10, 20, 30, and 40 wt% Al powder, respectively). A similar nomenclature was adopted for the samples containing RDX filler (see Table 2).

All the rheological measurements were performed on a Haake MARs Modular Advanced Rheometer System (Vreden, Germany) equipped with a 20 mm parallel plate geometry and a gap width of approximately 1 mm in air. The temperature was controlled with a Haake test chamber controller with an accuracy of ±1 °C. RheoWin Data Manager was used for equipment control, data acquisition, and treatments.

Dynamic temperature sweep experiments were performed at 1 rads^−1^ from 50 to 150 °C during heating at 2 °C min^−1^. Dynamic frequency sweep experiments were also conducted at a strain amplitude of 1% as a function of angular frequency (*ω*) ranging from 0.1 to 100 rad s^−1^. Strain sweep tests were performed from 0.1% to 100% strain at 1 rad s^−1^. Specimens were kept at a constant temperature for about 10 min before rheological measurements were obtained again. The temperature control was accurate to within ±1 °C, and a shear strain of 1% (region of linear viscoelastic response, obtained by means of a strain sweep experiment at 110 °C) was used.

## 3. Results

### 3.1. Influence of Al Powder Content on the Rheological Properties of the Model Propellants

#### 3.1.1. Influence of Al Powder Content on the Temperature Dependence of the Model Propellants

**Figure 1:**
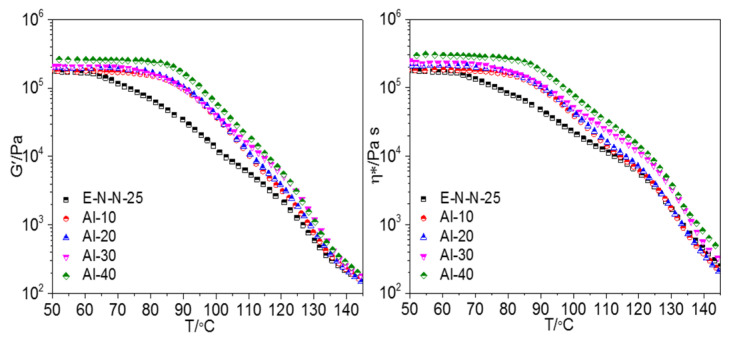
Temperature dependencies of (**left**) storage modulus G’ and (**right**) complex viscosity η* of model propellants with different Al contents.

Figure 1 shows the storage modulus and complex viscosities of the model propellants with different Al powder contents (whose morphology is shown in the Figure S1 of Supplementary Materials) as a function of temperature T. Both storage modulus and complex viscosities decreased with the increasing temperature throughout the testing range. This trend can be explained by the interaction of the hydroxyl radical groups on the surfaces of the Al powders [13] with the carbonyl groups of the ETPE and nitro groups of the NC in the adhesive [14]. The resultant hydrogen bonds attached to the adhesive molecules to the surfaces of the Al particles, hindering their molecular motions. Increasing the Al powder content in the model propellant increased the hydrogen-bond interaction between the Al powder and the adhesive. This can be confirmed by the changes of the crosslinking density of model propellants shown in the Figure S3 of the Supplementary Materials. Concretely, the curves could be divided into three stages according to the variation of slopes.

In the first stage, the storage modulus and complex viscosities of the model propellants decreased slowly with the increasing temperature, which was named as “platform zone”. In this stage, the temperature was below the glass transition temperature T_g_ of the propellants, and the adhesive was in a rubbery state, in which the segment motion was restricted. The temperature range of the platform zone increased with the increasing Al powder content because more Al powders started to interact with the adhesives. This interaction prevented motions of the hard segments of ETPE, thereby increasing the T_g_ of the model propellants. Accordingly, the platform moved toward the high-temperature area.

In the second stage, both the storage modulus and complex viscosities decreased rapidly with the increasing temperature. This may be because that the temperature of this stage approached the T_g_ of ETPE, so the movement of the hard segments of ETPE disrupted the physical crosslinking points (e.g., hydrogen bonds) which formed by the hard segments within the accumulation area of ETPE. As a result, the enhanced molecular motion of the adhesive could increase mobility of the unit (main chain segment of ETPE). Accordingly, the change of molecular conformation in the adhesive and the reduced deformability resistance of the model propellants accelerated the decrease rates of the storage modulus and complex viscosities.

In the third stage, the storage modulus and complex viscosities exhibited an even faster decrease with the increasing temperature. The chain segments of the NC molecules in the adhesive system began to move, and parts of the ETPE molecular chain segments underwent slippage. Consequently, the molecular motion in the adhesive system increased, and the molecular conformation altered.

**Figure 2:**
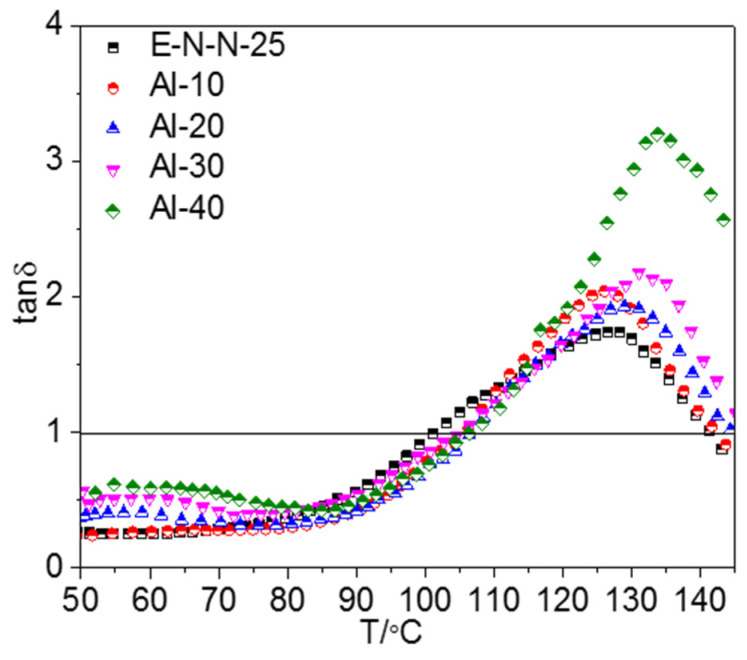
Loss factor (tan *δ*) versus temperature curves of model propellants with different Al contents.

The tan *δ*–T curves of the model propellants with different Al contents (tan *δ* refers to the loss factor) can be found in Figure 2. Similarly, the tan *δ*–T curves of the Al-based model propellants could be divided into three stages.

In the first stage, tan *δ* of the model propellant changed gradually with the increasing temperature. At low temperatures, the molecules of adhesive remained in a highly elastic state, and the mobile unit of the adhesive system was small, so the energy loss was low. As the Al content increased, the loss factor of the model propellant increased because Al formed hydrogen bonds with adhesive molecules, which increased the drag on the mobile unit of the adhesive and consequently the energy loss. This is consistent with the changes in mechanical properties of the Al-based model propellants shown in Table S1 of the Supplementary Materials.

The second stage was marked by a steep increase in tan *δ* because many physical crosslinking points in the adhesive system were damaged, increasing the number of mobile units in the system. The large-size mobile units were greatly blocked during the moving process, causing a noticeable increase in tan *δ*.

In the third stage, the tan *δ* decreased, but the number of mobile units of the adhesive increased from that of the second stage. The free volume of adhesive molecules increased with increasing temperature in this stage, which reduced the blocking effect on the mobile unit during the moving process and hence decreased the energy loss. Increasing Al content enhanced tan *δ*, indicating that adhesive molecules bonded to the surface of the Al powder through hydrogen-bond and Van der Waals (VDW) effect. As in the second stage, the increased drag on the mobile unit of the model propellant increased the energy loss.

Moreover, it is worth noting that the model propellants exhibited elastic characteristics until the temperature reached T_tan *δ*_ (the temperature at which tan *δ* becomes 1.0 and the model propellant transforms from an elastic to a viscous material). It could be found from Table 3 that T_tan *δ*_ was an increasing function of Al powder content in the propellant. T_tan *δ*_ showed a maximum value at T_g_, which shifted toward higher temperature as the Al powder content increased (Table 3). The change should arise from the increase in drag on the mobile unit of the model propellant, which caused by the hydrogen-bonding interaction between the adhesive and the Al powder Consequently, the energy input for transforming adhesive from elastic to viscous increased, and the characteristic temperature increased accordingly.

#### 3.1.2. Influence of Al Content on the Frequency Dependence of the Model Propellants

**Figure 3:**
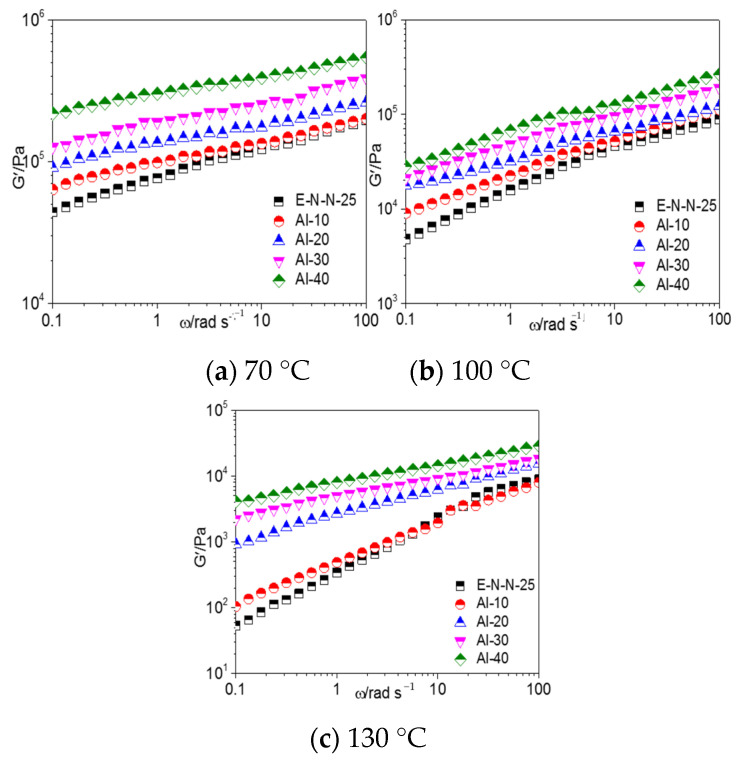
G′–*ω* curves of model propellants with different Al powder contents.

Figure 3 shows the G′–*ω* curves of the model propellants with different Al contents at different temperature. G′ of the model propellant was a decreasing function of temperature but an increasing function of frequency. Moreover, G′ of the Al-based model propellant increased with the increasing Al content with the constant temperature, which fully demonstrated the enhancement effect of the Al powder as a reinforcement material.

The variation amplitude of the storage modulus increased with the increase in Al content. This effect was stronger at lower temperatures owing to the enhanced mobility of the adhesive in the model propellant and the partial dissociation of hydrogen bonds between adhesive molecules and the Al powder.

With the increase in frequency, the variation amplitude of G′ with Al content decreased. In the model propellant, the adhesive molecules were absorbed on the surface of the Al powders, resulting in a stronger binding effect on the main chain segment of the adhesive molecule than that on the small units (e.g., chain links and side groups). Therefore, the influence of Al powder content on the model propellant is more significant in the long time zone (low-frequency zone).

**Figure 4:**
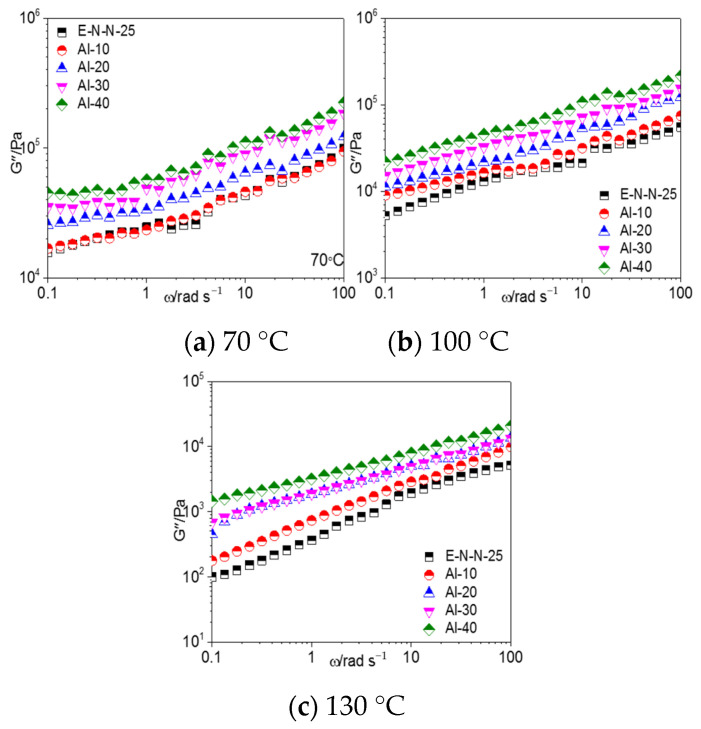
G″–*ω* curves of model propellants with different Al powder contents.

The frequency dependence of the loss modulus (G″) of the model propellants with different Al powder contents at different temperatures is shown in Figure 4. G″ of the model propellant increased with the increasing frequency and Al powder content but decreased with the increasing temperature.

**Figure 5:**
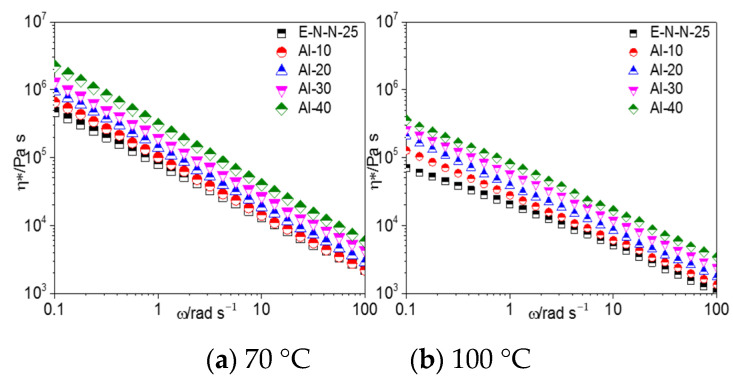

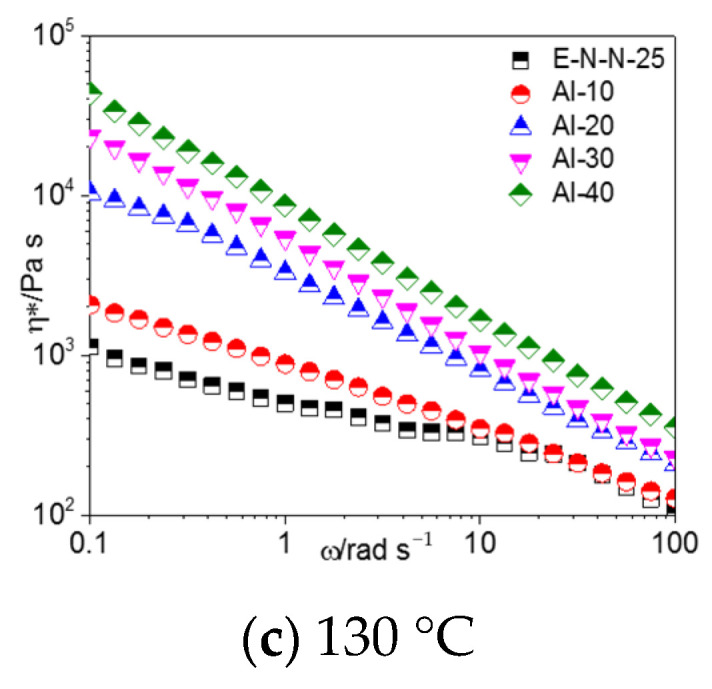
η*–*ω* curves of model propellants with different Al powder contents.

Figure 5 shows the η*–*ω* curves of model propellants with different Al contents. The Al-based model propellant exhibited the typical characteristics of pseudoplastic fluids. Similar to adhesive systems, the complex viscosity of the model propellant was a decreasing function of temperature and an increasing function of the Al content. These behaviors are explained by the aforementioned crosslinking phenomena between the adhesive and Al powder.

### 3.2. Influence of RDX Content on the Rheological Properties of the Model Propellants

#### 3.2.1. Influence of RDX Content on the Temperature Dependence of the Model Propellants

**Figure 6:**
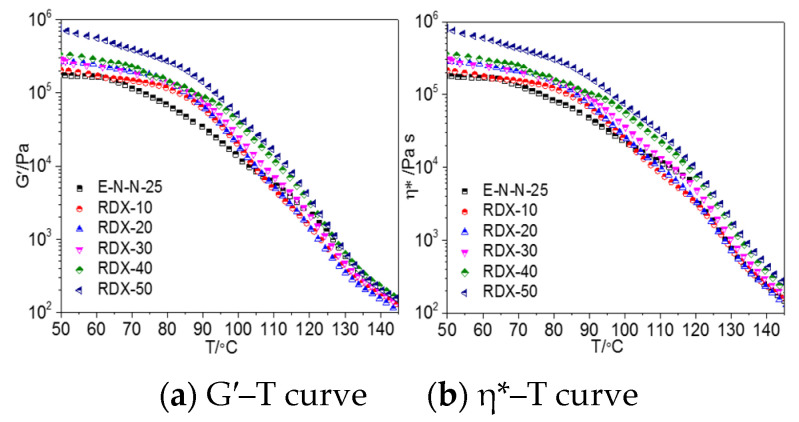
G′–T and η*–T curves of model propellants with different RDX contents (*γ* = 1%, *ω* = 1 rad/s).

Figure 6 shows the storage modulus and complex viscosities of the model propellants with different RDX contents whose morphologies are shown in Figure S2 of the Supplementary Materials. Both storage modulus and complex viscosities continuously decreased as the temperature increased. Similarly, these curves could be divided into three stages.

In the first stage, G′ and η* decreased slowly with the increasing temperature because the model propellant was elastic and the mobile units of the adhesive were small. In this stage, the molecular conformation of the adhesive could not change easily. The G′ and η* declined faster in RDX-based model propellants than that in the Al-based model propellants for the following reasons. Firstly, Al powder has larger specific surface area compared with RDX particles, which could provide larger contact area with the adhesive. Secondly, the wettability between Al powder and adhesive exceeded that of RDX, which resulted in a more fragile interaction between the RDX and adhesive. Therefore, the molecular interaction between Al powder and adhesive was significantly stronger than that of RDX. This is consistent with the changes in mechanical properties of the model propellants shown in Tables S1 and S2 of the Supplementary Materials.

The second and third stages were characterized by fast decreases in the G′ and η*. In these stages, the physical crosslinking points in the adhesive were damaged, and the molecular mobility was enhanced. As the number of mobile molecular units of the adhesive increased, the molecular conformation of adhesive was more easily altered, and G′ and η* increased more rapidly with increasing temperature. However, the G′ and η* of the propellant were lower than those of adhesive when the temperature increased to 100 °C. This phenomenon could be explained by the weakening of the physical crosslinking effect between the RDX and adhesive molecules at high temperatures and easy separation of RDX from the adhesive due to poor wettability. Moreover, at high temperatures, RDX can be partially dissolved in the polar plasticizer NENA, forming a soft lubricating layer on the contact interface between the RDX and adhesive. The lubricating effect of this layer reduced G′ and η* of the model propellants.

**Figure 7:**
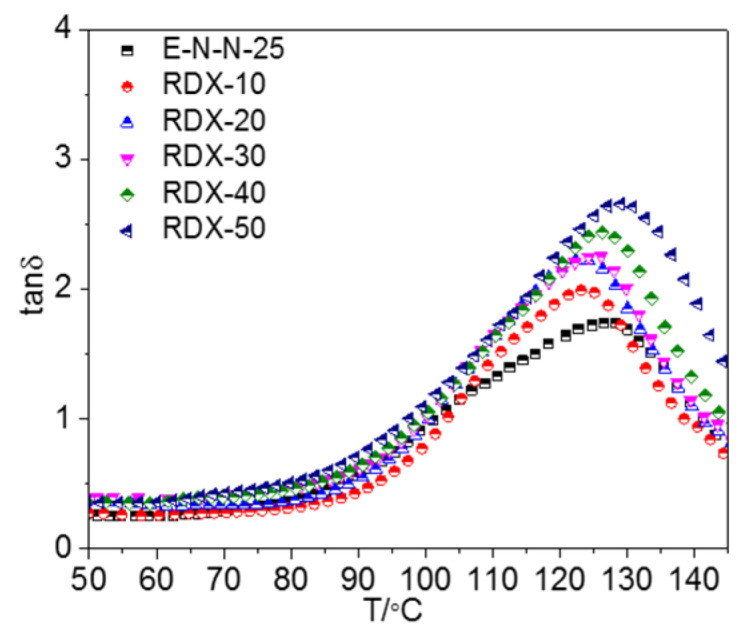
tan *δ*–T curves of model propellants with different RDX content (*γ* = 1%, *ω* = 1 rad/s).

Figure 7 shows the tan *δ*–T curves of the model propellants with different RDX contents, which could be also divided into three stages. In the first stage, tan *δ* of the model propellant changed slightly with the increasing temperature. The adhesive mainly retained its highly-elastic state, and the mobile unit of the adhesive system was small, incurring small energy loss. However, the energy loss of the model propellant increased with the increasing RDX content in this stage because the nitro groups of RDX were hydrogen-bonded to the hydroxyl groups of the adhesive molecules [15,16], which can also be confirmed by the changes of the crosslinking density of the model propellants shown in Figure S4 of the Supplementary Materials. This effect increased the energy loss and dragged the mobile unit in the adhesive. In the second stage, the loss factor of the model propellant began to increase. The physical crosslinking sites formed through the participation of ETPE hard segments were damaged, increasing the number of mobile units in the system and the energy loss of large-size mobile units, resulting in increasing tan *δ*. In the third stage, tan *δ* decreased because NENA could damage the entanglement sites in the adhesive molecules at high temperatures, resulting in slippage of ETPE molecules and an increase in the number of mobile molecular units. The increased molecular free volume of the adhesive reduced the blocking effect on the mobile units, thereby reducing the energy loss. However, tan *δ* of the model propellant increased after adding RDX because the adhesive was absorbed onto the surfaces of the RDX particles. The resulting hydrogen-bond interaction and VDW effect increased the drag on the mobile molecular unit of the adhesive, thus increasing tan *δ*.

Moreover, T_g_ showed a decrease followed by an increase with the increasing RDX contents (Table 4). This behavior might be due to the large interfacial tension between RDX and the adhesive and poor wettability in RDX-based model propellants, which facilitated the separation of RDX from the adhesive. As the temperature increased, the physical crosslinking effect between adhesive and RDX was weakened, and thus the RDX and adhesive were easily separated. While at high temperatures, RDX can be partially dissolved in the polar plasticizer, forming a soft layer on the contact interface between RDX and adhesive, as discussed above. The lubrication effect of this soft layer facilitated the movement of the adhesive molecules [17], thereby decreasing the T_g_.

#### 3.2.2. Influence of RDX Content on the Frequency Dependence of the Model Propellants

**Figure 8:**
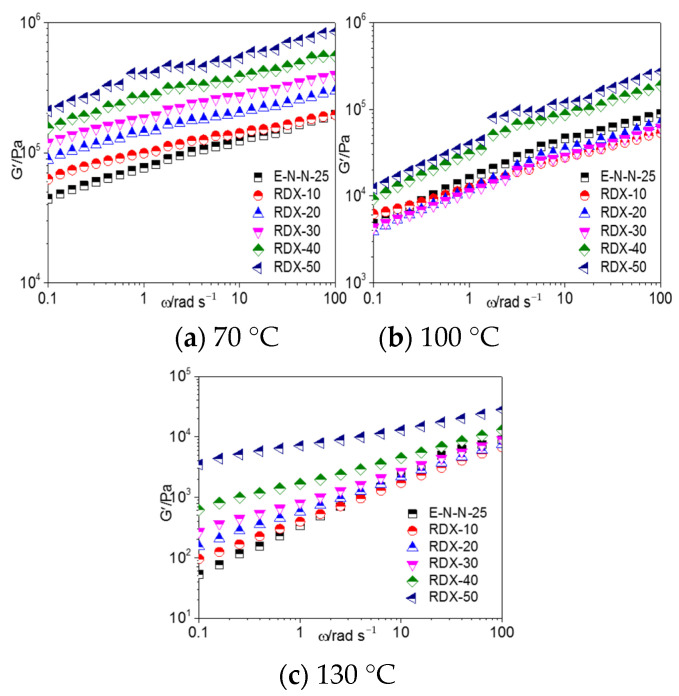
G′–*ω* curves of model propellants at (**a**) 70 °C, (**b**) 100 °C, and (**c**) 130 °C.

**Figure 9:**
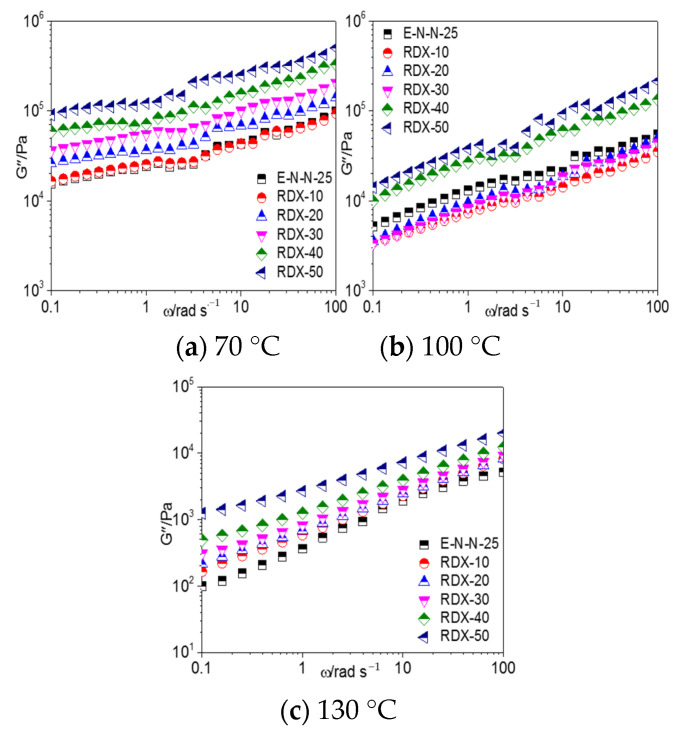
G″–*ω* curves of model propellant at (**a**) 70 °C, (**b**) 100 °C, and (**c**) 130 °C.

**Figure 10:**
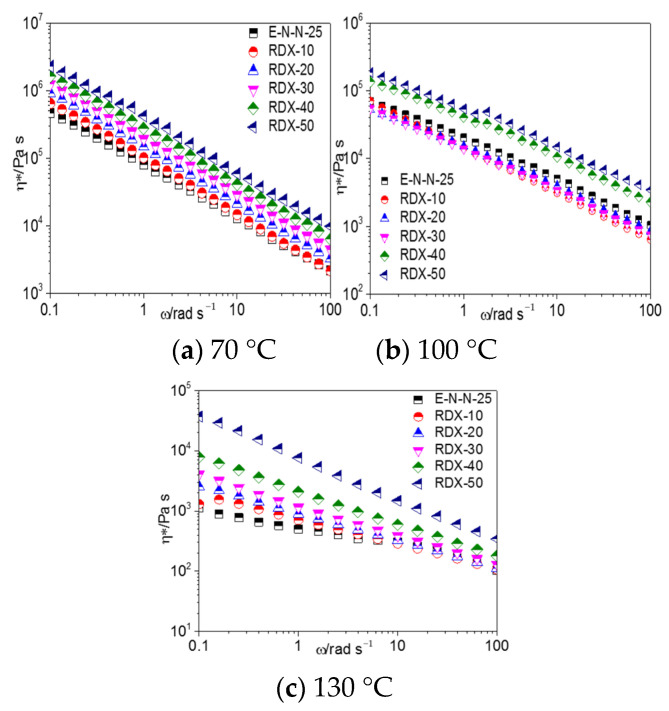
η*–*ω* curves of model propellants at (**a**) 70 °C, (**b**) 100 °C, and (**c**) 130 °C.

Figure 8, Figure 9, and Figure 10 show the G′–ω, G″–*ω* and η*–*ω* curves of the ETPE/NC/NENA/RDX model propellants, respectively, with different RDX contents at different temperatures. As shown, the storage modulus, loss modulus, and complex viscosities of the ETPE/NC/NENA/RDX model propellants decreased with the increasing temperature and increased with the increasing filler content. The effect of RDX content on the model propellant was weaker in the high-frequency region.

When the temperature increased to 100 °C, the effect of RDX content on the rheological parameters of the RDX-based model propellants differed from those at 70 °C. When the RDX content was lower than 30%, G′, G″, and the complex viscosity were smaller in the RDX-based model propellants than in the blank adhesive system. The high temperature increased the mobility of the mobile molecular units in the adhesive; consequently, the hydrogen-bond interactions between the nitro groups of the RDX and hydroxyl groups of the adhesives were disrupted, and a lubricating layer was formed as we discussed earlier. In contrast, when the RDX content exceeded 30%, the interactions between RDX and the adhesive molecules were enhanced, resulting the increasing G′, G″, and complex viscosity of the model propellants.

### 3.3. Application of Two-Phase Model in Model Propellants

As known, filler particles are dispersed throughout the polymer matrix. Deformation caused by the filled polymer system mainly influenced the matrix under external force, resulting in the local strain of the matrix far greater than the macrostrain and thus yielding strain amplification effect. This could be used to explain the increase in the modulus of the filled polymer [18]. The strain amplification factor Af may be measured via experiment at low filler content [19], and the Einstein equation, Guth–Gold equation, and their modifications can be used to describe the hydrodynamic strain amplification of particles [20,21]. However, fillers are found throughout the polymer matrix at high filler content, and the filler particles contact with each other or with polymers to form a network structure, invalidating the Einstein equation, Guth–Gold equation, etc. To study the viscoelastic behavior of the highly-filled polymer, Zheng et al. proposed a two-phase model to describe the viscoelastic behavior of the highly-filled polymer, in combination with the strain amplification effect of the polymer matrix phase and the viscoelastic contribution of filler particle phase [22,23].

**Figure 11:**
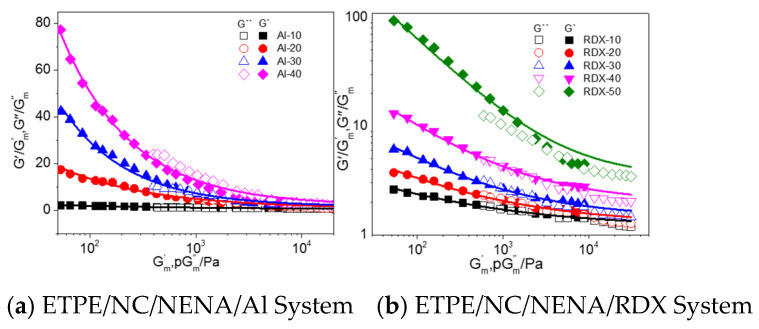
Relationship between $G^{\prime }\left(\omega ,\varphi \right)/G_{m}^{\prime }\left(\omega \right)-G_{m}^{\prime }\left(\omega \right)$ (filled dot) and $G^{\prime \prime }\left(\omega ,\varphi \right)/G_{m}^{\prime \prime }\left(\omega \right)-pG_{m}^{\prime \prime }\left(\omega \right)$ (hollow dot) of solid-filled model propellant at 130 °C.

Accordingly, the viscoelastic behavior of model propellants was studied with the multi-component adhesive system as matrix and Al powders or RDX particles as fillers. For example, the strain amplification and other factors were calculated by Equations (1)–(5) for Al-based and RDX-based model propellants at 130 °C, where $A_{f}\left(\varphi \right),$ $R_{f}^{\prime }\left(\varphi \right)$ and n were calculated by $G^{\prime }\left(\omega ,\varphi \right)/G_{m}^{\prime }\left(\omega \right)-G_{m}^{\prime }\left(\omega \right)\;\mathrm{and}\;G^{\prime \prime }\left(\omega ,\varphi \right)/G_{m}^{\prime \prime }\left(\omega \right)-pG_{m}^{\prime \prime }\left(\omega \right)$, respectively (Figure 11, Table 5).

(1) $$G^{*}\left(\omega ,\varphi \right)=A_{f}\left(\varphi \right)G_{m}^{*}\left(\omega \right)+G_{f}^{*}\left(\omega ,\varphi \right)$$

where $G^{*}\left(\omega ,\varphi \right)$, $G_{m}^{*}\left(\omega \right),$ and $G_{f}^{*}\left(\omega ,\varphi \right)$ are the complex modulus of the filled polymer, the polymer matrix and the filler particle, respectively; *A_f_* is the strain amplification factor. The complex modulus of the filler particle may be obtained by the microrheological model [23,24].

(2) $$G_{f}^{*}\left(\omega ,\varphi \right)={\left[G_{m}^{\prime }\left(\omega \right)\right]}^{n}+iR_{f}^{\prime \prime }\left(\varphi \right){\left[G_{m}^{\prime \prime }\left(\omega \right)\right]}^{n}$$

where $R_{f}^{\prime }\left(\varphi \right)$ and $R_{f}^{\prime \prime }\left(\varphi \right)$ refer to the elastic and viscous contribution of filler particles, respectively, between which the relationship is as follows:

(3) $$R_{f}^{\prime \prime }\left(\varphi \right)=R_{f}^{\prime }\left(\varphi \right)p^{n-1}$$

where *p* is a dimensionless parameter related to the degree of aggregation of filler particles and the polymer matrix–filler particle interface interaction. 

The two-phase model was used to discuss the linear viscoelasticity of the highly-filled polymer system. The combinations of Equations (1)–(3) were transformed into

(4) $$G^{\prime }\left(\omega ,\varphi \right)/G_{m}^{\prime }\left(\omega \right)=A_{f}\left(\varphi \right)+R_{f}^{\prime }\left(\varphi \right){\left[G_{m}^{\prime }\left(\omega \right)\right]}^{n-1}$$

(5) $$G^{\prime \prime }\left(\omega ,\varphi \right)/G_{m}^{\prime \prime }\left(\omega \right)=A_{f}\left(\varphi \right)+R_{f}^{\prime }\left(\varphi \right){\left[pG_{m}^{\prime \prime }\left(\omega \right)\right]}^{n-1}$$

**Figure 12:**
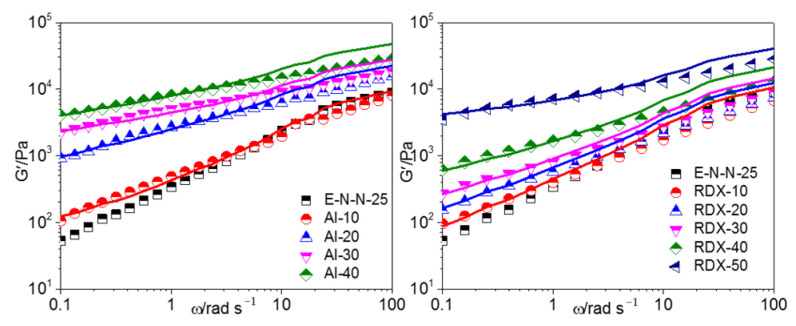
Measured values in dot and two-phase model simulation values in solid line under the curve G′-*ω* of model propellant at 130 °C.

**Figure 13:**
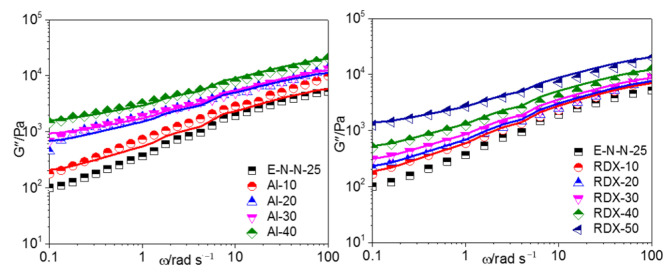
Measured values in dot and two-phase model simulation values in solid line under the curve G″-*ω* of model propellant at 130 °C.

The G′-*ω* and G″-*ω* curves of the model propellants at 130 °C are shown in Figure 12 and Figure 13, respectively, where the measured values are shown in dots and the simulation values in solid lines. It could be found that the two-phase model may well describe the linear viscoelastic behavior of the two model propellants at low frequency, while deviation is found in the high frequency range. This may be because the strain amplification effect of fillers on adhesive molecules should be deemed as constant for the two-phase model. However, kinematic units of actual model propellants are segments of the adhesive molecule backbone in low frequency range, the filler particles showed a significant binding effect and a considerable strain amplification effect on the adhesive molecules due to the adsorption of filler particles by adhesive molecules. In high frequency range, kinematic units of adhesives were the pendant groups and chain links of adhesive molecules, but there is poor interaction between the filler particles and the kinematic units, exerting a slight effect on the kinematic units, resulting in the declined strain effect.

**Figure 14:**
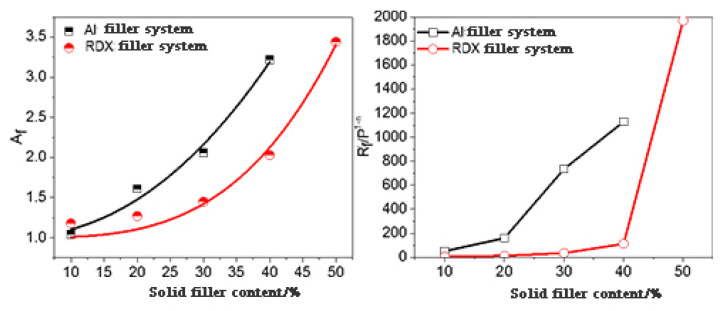
Changes of two-phase model parameters with the content of solid fillers.

Figure 14 shows that how the strain amplification factor *A_f_* and the elastic contribution $R_{f}^{\prime }\left(\varphi \right)$ of the Al-based and RDX-based model propellants changed with the solid particle content, and the two-phase model parameters are listed in Table 5. It can be found that *A_f_* is exponentially dependent on the content of solid particles in the blend system, and the *A_f_* of the model propellants rapidly rises with the increasing solid filler content. Moreover, $R_{f}^{\prime }$ grows with the solid content, increasing the contribution of solid particles to the viscoelastic parameters of the model propellants.

Due to the high solid content, the Einstein equation, the Guth–Gold equation, and other equations are invalid to study the relationship between *A_f_* and solid content. The Huber–Vilgis equation was adopted to fit the relationship between *A_f_* and solid content. The Huber–Vilgis equation is as follows:

(6) $$A_{f}\left(\varphi \right)=1+C\times {\left(a\times \varphi \right)}^{\frac{2}{3-d_{f}}}$$

where a is an effective volume fraction of particles, C is an aggregate variable related to the primary particle size, the aggregate size, and the irregular diffusion index of a the ggregate. $d_{f}$ is a sub-dimension representing the size of the particle aggregate, which is 1.80 as theoretically predicted and measured at a value between 1–3 for different systems.

(7) $$A_{f}\left(\varphi \right)=1+2.4\times {\left(2.4\varphi \right)}^{\frac{2}{3-2.1}}$$

(8) $$A_{f}\left(\varphi \right)=1+2.1\times {\left(2.1\varphi \right)}^{\frac{2}{3-2.4}}$$

According to the fitted relational expression, a of the Al-based model propellant is greater than that of RDX-based. This means that the effective volume of Al powders in the propellant is greater, indicating that Al powders are evenly dispersed in the adhesive system. The Al-based model propellant exhibited a greater $d_{f}$ than the RDX-filled system, indicating that the aggregation of Al powders is slight in the corresponding model propellant [25]. Meanwhile, it could be found that Al powders make greater contributions to the adhesive system than RDX particles through comparison between two solid fillers: the *A_f_* and $R_{f}^{\prime }\left(\varphi \right)$ of Al powders are larger than RDX particles. That is why Al-based model propellants showed the larger rheological parameter.

**Figure 15:**
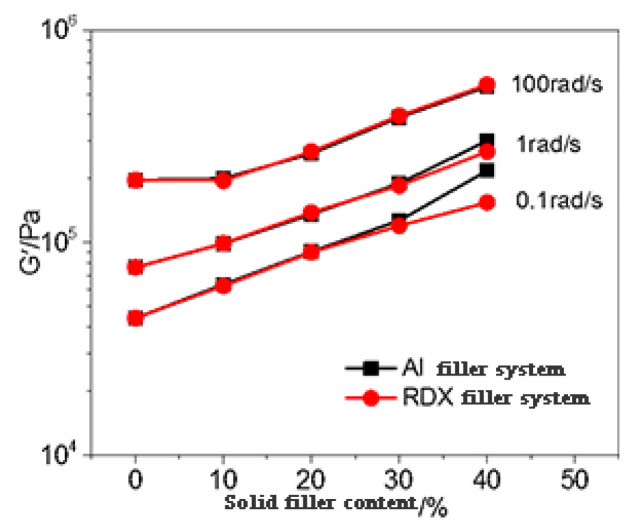
Relationship between G′ of solid-filled model propellants and filler content at different angular frequencies.

Figure 15 shows the relationship between G′ of the two model propellants and the filler content at different frequencies. The G′ of the Al-based model propellant is approximately equal to that of the RDX-based model propellant when the solid filler content is lower than 20% and where G′ values are different at different frequencies when the mass fraction is higher than 20%. Moreover, the two model propellants have almost the same G′ at 100 rad/s, where the G′ of the Al-based model propellant is greater than that of the RDX-based at 10 rad/s when the filler content is 30%, and the two are different when the content rises to 40%, and the difference is much more considerable at 0.1 rad/s.

This may be because there are three kinds of interactions in the model propellants [26]: the interactions among polymer matrix molecules, between filler particles and polymer molecules, and among filler particles. When the dosage of fillers is low, solid fillers could be evenly dispersed in the adhesive system. Accordingly, it is almost not likely to find any interaction among particles, resulting in the three types of interactions making few contributions to the G′ of the model propellants, which results in a slight difference between the two model propellants in G′. Such interactions are enhanced when the content of fillers increases, contributing to larger internal forces in all of the model propellants. Both the strain amplification factor and the elastic contribution of fillers exponentially grow, while that of the Al-based model propellant grows faster. In the high-frequency range, the model propellant adhesive has small kinematic units on which there is a slight effect of fillers, so the G′ of the two model propellants tends to coincide.

## 4. Conclusions

In this paper, two novel model propellants, ETPE/NC/NENA/Al and ETPE/NC/NENA/RDX, were fabricated based on an ETPE/NC/NENA adhesive matrix and Al powder or RDX filler. The influence of solid filler on the rheological properties of the model propellants was investigated and a corresponding two-phase model was established. The content of both Al powder and RDX slightly affected the temperature effects of the viscoelastic parameters G′, G″, and η* of the model propellants. The modification was significantly observed in the third stage of the viscoelastic parameter versus temperature plots. At a given temperature, G′, G″, and η* increased with the increasing filler content. It is noteworthy that, when the temperature increased to 100 °C or higher, the viscoelastic parameters (G′, G″, and η*) of ETPE/NC/NENA/RDX with an RDX content below 30% were lower than those of the corresponding adhesive. The two-phase model well described the linear viscoelastic behavior of the Al-based and RDX-based model propellants in the low frequency range. In high solid contents, the viscoelastic behavior of the model propellants is affected by both the adhesive matrix and the solid fillers. By comparing two model propellants, it can be concluded that when the strain amplification factor *A_f_* and the elastic contribution $R_{f}^{\prime }\left(\varphi \right)$ of the Al-based model propellant are greater than those of the RDX system, *A_f_* and $R_{f}^{\prime }\left(\varphi \right)$ exponentially grow with the content of solid fillers, and *A_f_* and $R_{f}^{\prime }\left(\varphi \right)$ of the Al powder system grow faster than the RDX system. The results may provide a reference for the structural design, molding process, and parameter selection of high-performance GAP-based green solid propellants.

## Figures and Tables

**Table 1:** Model propellants with different Al contents.

Samples	Adhesive (ETPE/NC/NENA) (wt%)	Al (wt%)
Al-10	90	10
Al-20/C-Al-20	80	20
Al-30	70	30
Al-40	60	40
A-Al-20	80	20
B-Al-20	80	20

**Table 2:** Model propellants with different RDX contents.

Samples	Adhesive (ETPE/NC/NENA) (wt%)	RDX (wt%)
RDX-10	90	10
RDX-20	80	20
RDX-30	70	30
RDX-40	60	40
RDX-50/C RDX-50	50	50
A-RDX-50	50	50
B-RDX-50	50	50

**Table 3:** Characteristic temperatures of the Al-based model propellants.

Samples	T_tanδ_ (°C)	T_g_ (°C)
E-N-N-25	101.2	125.4
Al-10	105.9	127.2
Al-20	105.9	128.0
Al-30	106.1	132.2
Al-40	106.8	135.1

**Table 4:** T_g_ values of RDX filler systems.

Samples	T_g_ (°C)
E-N-N-25	125.4
RDX-10	125.2
RDX-20	125.3
RDX-30	126.2
RDX-40	126.3
RDX-50	128.2

**Table 5:** Parameters of the two-phase model of model propellants.

Sample	$A_{f}\left(\varphi \right)$	$R_{f}^{\prime }\left(\varphi \right)$/Pa^1−n^	n
Al-10	1.04	51.8	0.35
Al-20	1.61	160.1	0.43
Al-30	2.06	735.4	0.23
Al-40	3.22	1128.4	0.23
RDX-10	1.18	6.1	0.65
RDX-20	1.27	14.3	0.58
RDX-30	1.45	35.4	0.51
RDX-40	2.03	112.4	0.43
RDX-50	3.44	1969.2	0.24

## Data Availability

Not applicable.

## Supplementary Materials

The following supporting information can be downloaded at: https://www.mdpi.com/article/10.3390/ma16020808/s1.

## References

[1] Tan H. (2015). The Chemistry and Technology of Solid Rocket Propellant.

[2] Mehta P.K., Kumaraswamy A., Saraswat V.K., Praveenkumar B. Recycling of Waste Propellant and the Challenges in Disposal: Range Safety. Proceedings of the 2021 2nd International Conference on Range Technology (ICORT).

[3] Dîrloman F.M., Toader G., Rotariu T., Țigănescu T.V., Ginghină R.E., Petre R., Alexe F., Ungureanu M.I., Rusen E., Diacon A. (2021). Novel Polyurethanes Based on Recycled Polyethylene Terephthalate: Synthesis, Characterization, and Formulation of Binders for Environmentally Responsible Rocket Propellants. Polymers.

[4] Wilkinson P.J., Weaver M.C., Kister G., Gill P.P. (2022). Styrene-Ethylene/Butylene-Styrene (SEBS) Block Copolymer Binder for Solid Propellants. Propellants Explos. Pyrotech..

[5] Shim Y.H., Ha S.W., Yoo E.S., Jang H.R., Kim A.R., Ramakrishnan S., Yoo D.J. (2021). Recovery of Ammonium Perchlorate from Obsolete Ammunition and Its Application in Synthesis of Lithium Perchlorate. Propellants Explos. Pyrotech..

[6] Jiu Y., Luo Y., Ge Z., Chai C., Xiao J., Zhang C. (2010). Studies on properties of ETPUEs containing PET and GAP as soft-segment. J. Solid Rocket Technol..

[7] Song X.D., Zhao F.Q., Xu S.Y. (2006). Combustion mechanism of double-base propellant containing bismuth 2,4-dihydroxylbenzoate. J. Propuls. Technol..

[8] Lv Y., Luo Y., Ge Z. (2008). Research development of energetic thermoplastic elastomers. New Chem. Mater..

[9] Mahanta A.K., Pathak D.D. (2010). Recent advances in development of eco-friendly solid composite propellants for rocket propulsion. Res. J. Chem. Environ..

[10] Luo Y., Ding S., Zhang C. (2022). Research Progress on Energetic Thermoplastic Elastomers. Mater. China.

[11] Chen M., Xu M., Liu N., Mo H., Lu X. (2020). Research progress of energetic thermoplastic adhesives. Explos. Mater..

[12] Shao Z., Wang F., Liao B. (2005). Homogeneous Synthesis of Azidodeoxycellulose. Chin. J. Explos. Propellants.

[13] Padhye R., Aquino A.J., Tunega D., Pantoya M.L. (2016). Effect of polar environments on the aluminum oxide shell surrounding aluminum particles: Simulations of surface hydroxyl bonding and charge. ACS Appl. Mater. Interfaces.

[14] Li B., Zhao Y., Li X., Luo Y. (2015). Synthesis and representation of random block type PBAMO/GAP ETPE. Chin. J. Energ. Mater..

[15] Wei H., Fu X., Deng C., Fan X., Zhou W., Li J. (2011). Surface and interfacial properties of star-class GAP and solid propellant filler. J. Solid Rocket Technol..

[16] Wang H., Fan X., Zhou W., Liu X., Wei H., Fan M. (2011). Surface and interfacial properties of reduced-smoke modified double base propellant with nitramine and Al powder. Chin. J. Explos. Propellants.

[17] Deng J. (2016). Research on GAP-Based High-Energy Solid Propellent.

[18] Liao Z., Hossain M., Yao X. (2020). Ecoflex polymer of different Shore hardnesses: Experimental investigations and constitutive modelling. Mech. Mater..

[19] Botti A., Pyckhout-Hintzen W., Richter D., Urban V., Straube E. (2006). A microscopic look at the reinforcement of silica-filled rubbers. J. Chem. Phys..

[20] Ahmed S., Jones F.R. (1990). A review of particulate reinforcement theories for polymer composites. J. Mater. Sci..

[21] Osman M.A., Atallah A., Schweizer T., Öttinger H.C. (2004). Particle-particle and particle-matrix interactions in calcite filled high-density polyethylene-steady shear. J. Rheol..

[22] Song Y., Zheng Q. (2010). Linear viscoelasticity of polymer melts filled with nano-sized fillers. Polymer.

[23] Song Y., Zheng Q. (2011). Application of two phase model to linear dynamic rheology of filled polymer melts. Polymer.

[24] Wolthers W., Van den Ende D., Breedveld V., Duits M.H., Potanin A.A., Wientjes R.H., Mellema J. (1997). Linear viscoelastic behavior of aggregated colloidal dispersions. Phys. Rev. E.

[25] Guan A. (2013). Conductive and Rheological Behavior of Multi-Walled Carbon Nanotube Filled with Polymer Composite System.

[26] Davris T., Mermet-Guyennet M.R., Bonn D., Lyulin A.V. (2016). Filler size effects on reinforcement in elastomer-based nanocomposites: Experimental and simulational insights into physical mechanisms. Macromolecules.

