# Supplementary material for: Influence of Solid Filler on the Rheological Properties of Propellants Based on Energetic Thermoplastic Elastomer
Source: Materials (Basel). 2023 Jan 13;16(2):808. doi: 10.3390/ma16020808 (PMC9862051; doi:10.3390/ma16020808)
Supplement: Supplementary file 1 [file materials-16-00808-s001.zip › materials-2124259-supplementary.pdf]

Supplementary material

Influence of Solid Filler on the Rheological Properties of Propellants Based on Energetic Thermoplastic Elastomer

Jing Zhang [1,2], Zhen Wang [1,2], Shixiong Sun [3,4] and Yunjun Luo [1,2,*]

[1] School of Materials Science and Technology, Beijing Institute of Technology, Beijing 100081, China
[2] Key Laboratory for Ministry of Education of High Energy Density Materials, Beijing 100081, China
[3] School of Chemistry and Chemical Engineering, North University of China, Taiyuan 030051, China
[4] Dezhou Industrial Technology Research Institute of North University of China, Dezhou 253034, China
* Correspondence: yjluo@bit.edu.cn

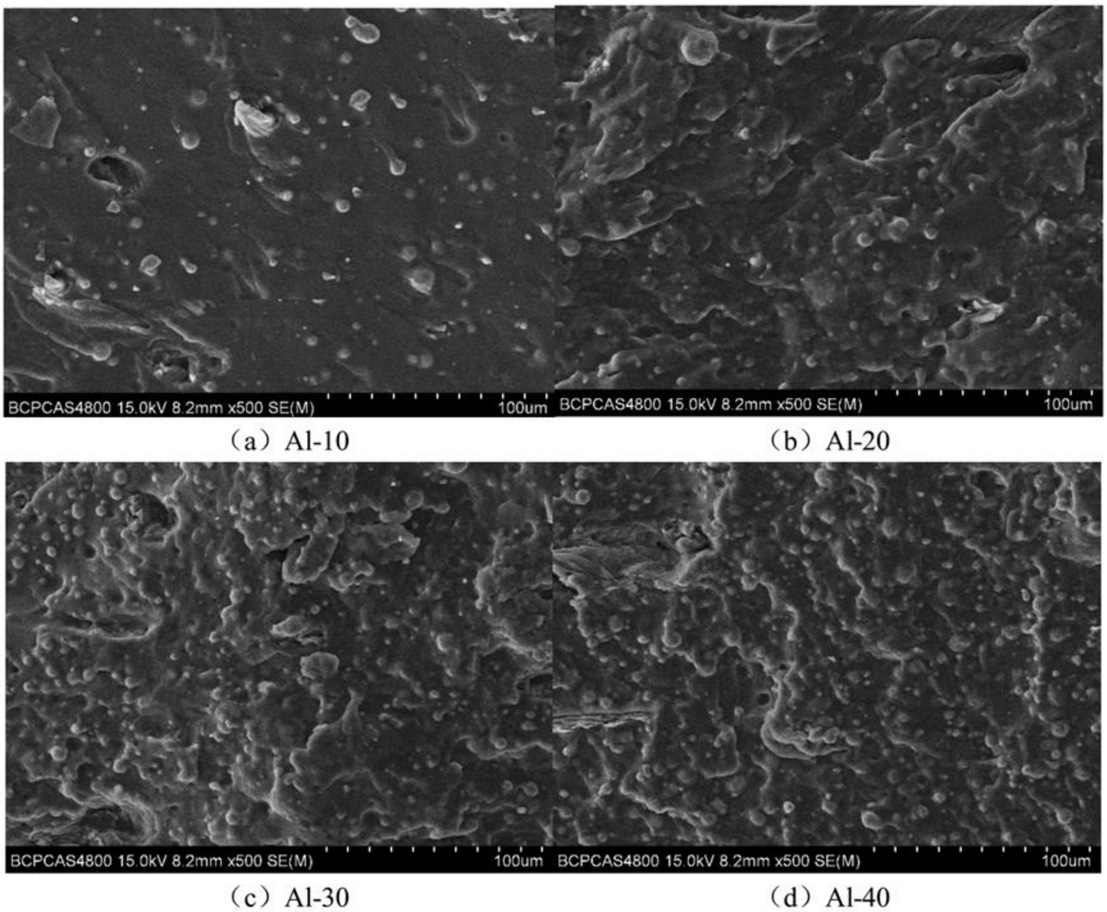

Figure S1. SEM image of model propellants with different Al contents.

With the increase of Al powder content, the Al powder particles on the surface of the model propellant increase. When the content of Al powder is 10%, the local surface of the model propellant is smooth. When the content of Al powder increases to 40%, a large number of Al powder particles are distributed on the surface of the model propellant, and the surface is rough. There is no obvious boundary between the contact interface of Al powder particles and the adhesive matrix, and the adhesion between Al powder particles and the adhesive is good.

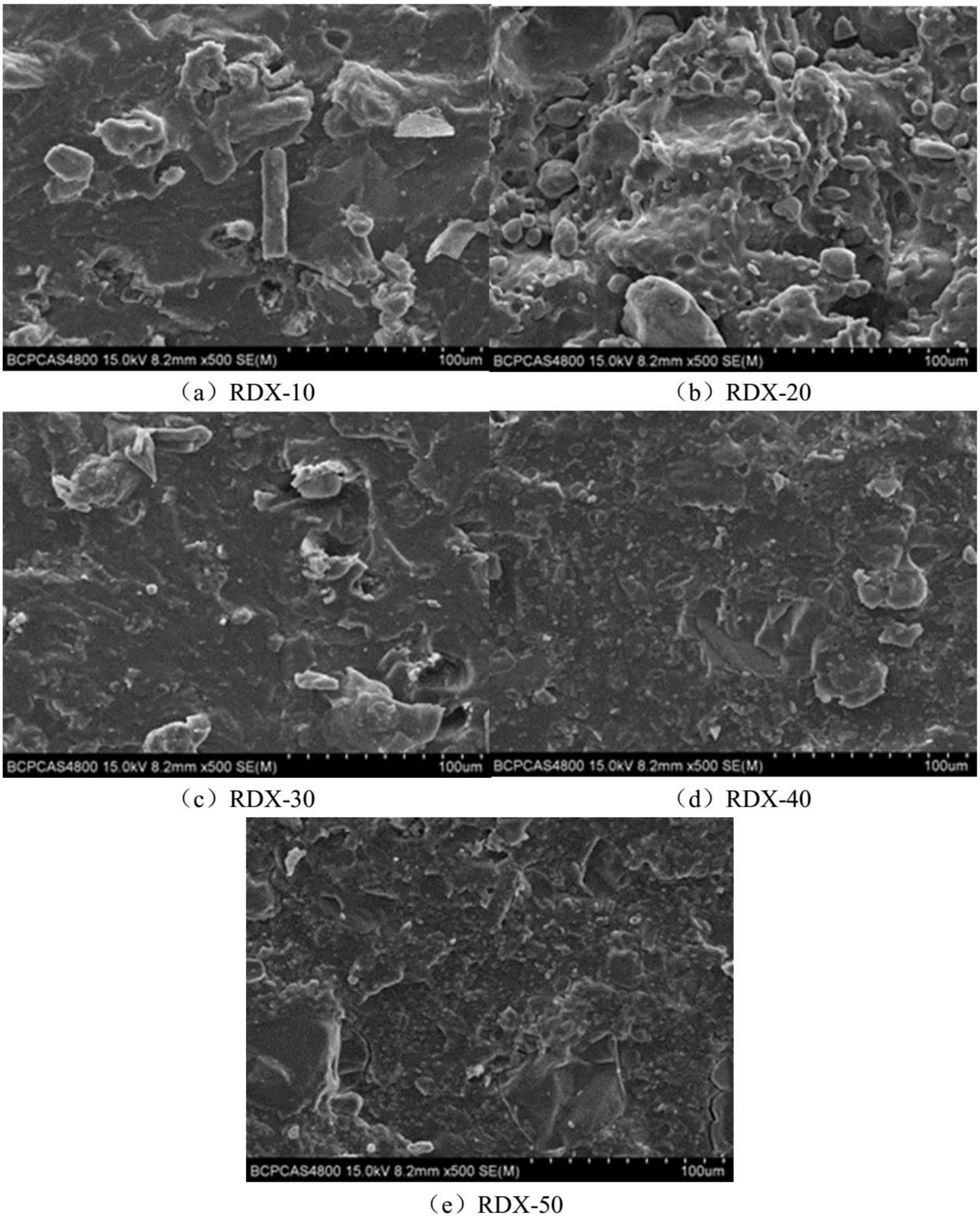

Figure S2. SEM image of model propellants with different RDX contents.

With the increase of RDX content, the RDX distribution on the surface of the model propellant becomes denser and denser. Due to the large particle size distribution of RDX particles, there are RDX particles with large particle sizes on the surface of the adhesive matrix. There is an apparent interface between RDX and the adhesive. In addition, there are obvious holes in the binder matrix, which are caused by the separation of RDX particles from the binder when the model propellant is cracked.

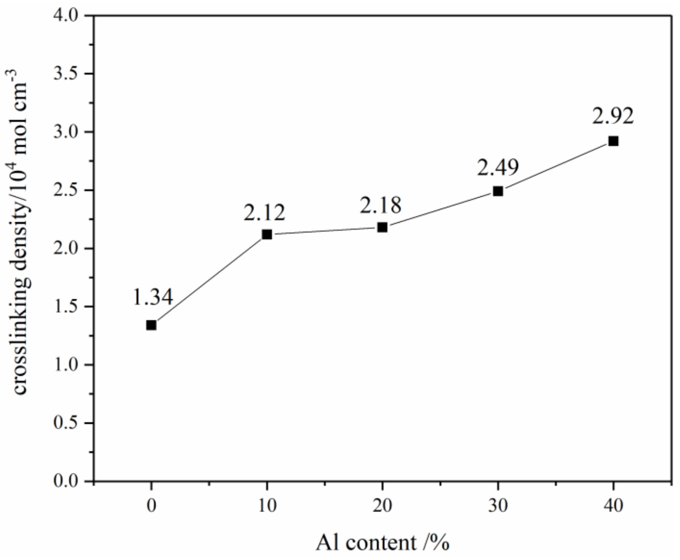

Figure S3. Crosslinking density of model propellants with different Al contents.

The crosslinking density of Al-based model propellant increases with the increase of Al powder content. Physical cross-linking points are formed by topological entanglement of EPTE and NC molecules, van der Waals force interaction between molecules, hydrogen bond interaction, and aggregation of hard segments with less content in ETPE in the model extruding agent adhesive system. With the addition of Al powder, the adhesive contacts with Al powder. At the contact position between Al powder and adhesive matrix, the hydroxyl groups on the surface of Al powder can form hydrogen bonds, van der Waals forces, and other effects with carbonyl groups in the adhesive molecules, so that the adhesive molecules are physically adsorbed on the surface of Al powder, and the Al powder acts as a physical cross-linking point.

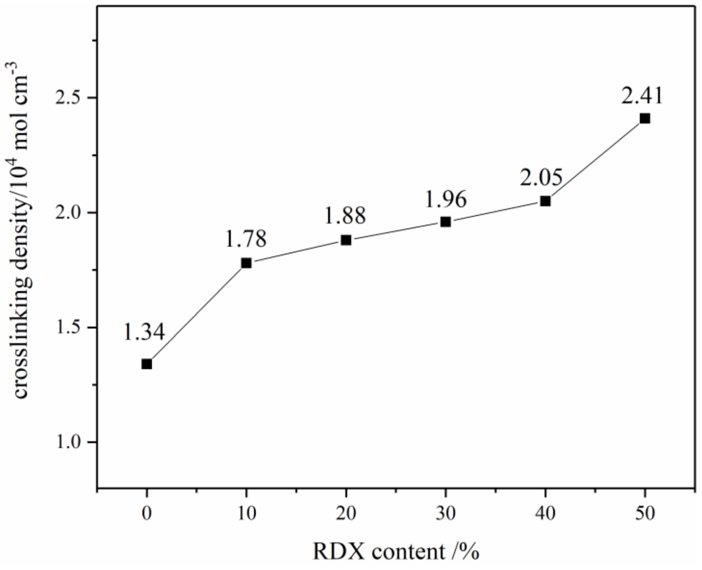

Figure S4. Crosslinking density of model propellants with different RDX contents.

The crosslinking density of RDX-based model propellant increases with the increase of RDX content. The nitro groups on the RDX surface can form hydrogen bonds with the hydroxyl groups in the NC molecules in the binder system, and can also induce the ester groups and azide groups in the ETPE molecules, so that the binder molecules are adsorbed

on the RDX particle surface, increasing the physical crosslinking points in the model propellant, and increasing the crosslinking density of the model propellant binder system.

Table S1. Mechanical property parameters of the Al-based model propellants.

Samples	σ/MPa	ε/%
E/N/N-25	3.50	152.1
Al-10	3.60	65.6
Al-20/C-Al-20	3.80	57.3
Al-30	3.90	48.4
Al-40	4.10	37.1
A-Al-20	3.52	63.1
B-Al-20	3.78	59.3

The tensile strength of the model propellant increases with the increase of Al powder content. This is because Al powder is a reinforcing material, which plays an obvious role in strengthening the adhesive system. With the increase of Al content, the physical adsorption between the binder and Al powder is stronger, which makes the physical crosslinking network structure of the model propellant more stable. When Al powder was mixed into the binder matrix, it contacted the molecular chain of the binder matrix and physical adsorption occurs, which blocks the movement of the molecular chain of the binder matrix and significantly reduces the elongation at break of the model propellant.

Table S2. Mechanical property parameters of the RDX-based model propellants.

Samples	σ/MPa	ε/%
E/N/N-25	3.50	152.1
RDX-10	3.10	58.2
RDX-20	2.40	57.4
RDX-30	2.10	48.0
RDX-40	2.00	36.4
RDX-50/C-RDX-50	2.30	29.3
A-RDX-50	1.70	25.1
B-RDX-50	2.10	25.4

RDX is a nonreinforced solid filler, which has a small interfacial adhesion with the adhesive system, and the wettability between them is relatively poor. RDX is easy to "detach" from the adhesive matrix when subjected to external forces.
